# Antiphospholipid antibody-associated cystic lesion of the pancreatic head with concurrent acute pancreatitis

**DOI:** 10.1515/rir-2025-0008

**Published:** 2025-04-02

**Authors:** Junxian Hong, Shikai Hu, Jiuliang Zhao, Yangzhong Zhou

**Affiliations:** Department of Rheumatology and Clinical Immunology, Chinese Academy of Medical Sciences and Peking Union Medical College, Beijing, China; School of Medicine, Tsinghua Medicine, Tsinghua University, Beijing, China; National Clinical Research Center for Dermatologic and Immunologic Diseases (NCRC-DID), Ministry of Science and Technology, Beijing, China; State Key Laboratory of Complex Severe and Rare Diseases, Peking Union Medical College Hospital (PUMCH), Beijing, China; Key Laboratory of Rheumatology and Clinical Immunology, Ministry of Education, Beijing, China

**Figure 1:**
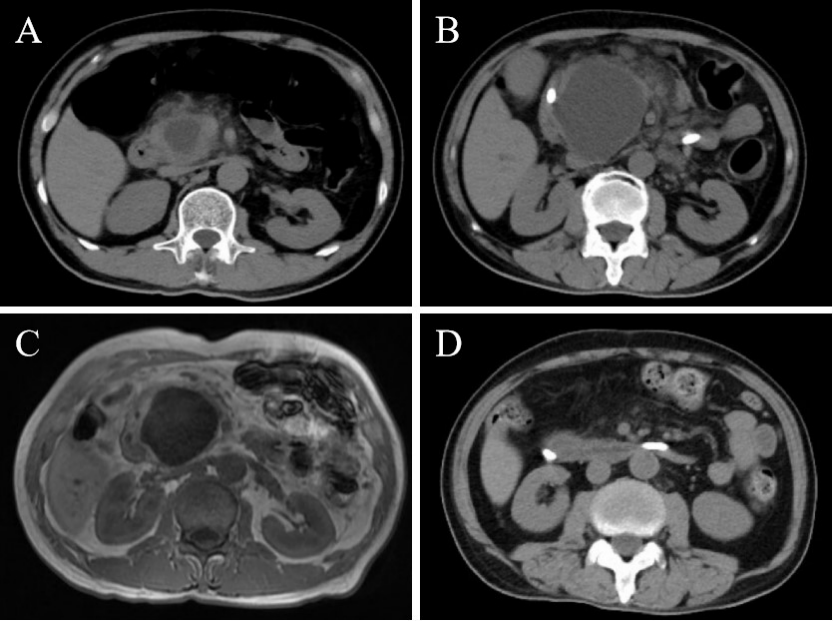
Images of the patient’s pancreas. Initial CT scan on admission (A) demonstrated a well-circumscribed cystic lesion (36 mm × 26 mm) within the pancreatic head, accompanied by peripancreatic exudation. CT scan after two weeks (B) showed progressive enlargement of the cyst to 67 mm × 49 mm, accompanied by dilation of the branch pancreatic ducts. Contrast-enhanced MRI (C) revealed hypointense on T1-weighted images and hyperintense on T2-weighted images within the pancreatic head, with no abnormal enhancement post-contrast administration. Follow-up CT scan after two weeks (D) demonstrated a significant reduction in cyst lesion size and resolution of peripancreatic exudation.

A 58-year-old male presented with an 8-day history of severe upper abdominal pain and vomiting. His medical history was unremarkable, with a normal abdominal computed tomography (CT) six months prior. Laboratory tests revealed elevated serum amylase, lipase, C-reactive protein, and D-dimer levels. CT scan identified a well-circumscribed 36 mm × 26 mm cystic lesion within the pancreatic head with peripancreatic exudation (Figure 1A). Despite supportive treatment for pancreatitis, symptoms persisted. Over two weeks, the lesion progressively enlarged to 67 mm × 49 mm on CT and magnetic resonance imaging (MRI), with branch pancreatic duct dilation (Figure 1B-C). Conventional etiologies such as biliary obstruction, hypertriglyceridemia, alcohol, drugs, or infection were excluded. An autoimmune workup revealed a normal immunoglobulin G4 (IgG4) level but positive antiphospholipid antibodies (aPL: Lupus anticoagulant (LA) 1.41 [positive: > 1.20]; anticardiolipin antibody (ACL)-IgM, 12.5 IgM phospholipid units (MPLU)/mL [positive: > 12.0]; beta-2 glycoprotein 1 (β2GP1)-IgM 60.1 AU/mL [positive: > 24.0]). The cystic lesion, inconsistent with intraductal papillary mucinous neoplasm (IPMN) or pseudocyst, led to the diagnosis of aPL-associated pancreatic cystic lesion with concurrent acute pancreatitis. Thromboembolic screening was negative. Methylprednisolone (40 mg/day) and low-molecular-weight heparin (4000 U/day) were administered, along with biliary and pancreatic duct stenting. As symptoms resolved, medications were tapered. Follow-up imaging demonstrated marked cyst regression (Figure 1D). Repeated testing after three months confirmed persistent aPL positivity (LA 1.28, ACL-IgM 14.9 MPLU/mL, β2GP1-IgM 42.6 AU/mL).

This case highlights a rare manifestation of patients with aPL positivity, as pancreatic involvement occurs in ~0.5% of antiphospholipid syndrome (APS) patients.^[1]^ While pancreatitis is common, aPL-associated cystic lesions are unusual but clinically significant.^[2, 3, 4]^ The proposed mechanism involves aPL-mediated microvascular thrombosis, leading to ischemic necrosis, inflammation, and cystogenesis.^[1,5]^ Persistent aPL positivity and therapeutic responsiveness further support aPLs as the etiology. This case underscores the importance of recognizing the diverse organ involvement and micro-vascular pathology of APS. Early identification and targeted treatment addressing inflammation and thrombosis can significantly benefit patients in such scenarios.
